# A systematic review of proteomic biomarkers in oral squamous cell cancer

**DOI:** 10.1186/s12957-021-02423-y

**Published:** 2021-10-28

**Authors:** Jyotsnaa Pillai, Tanvi Chincholkar, Ruhi Dixit, Manoj Pandey

**Affiliations:** 1 Datta Meghe Institute of Medical Sciences, Wardha, India; 2 Department of Surgical Oncology, Institute of Medical Sciences, Banaras Hindu University, Varanasi, 221 005 India

**Keywords:** Head and neck cancer, Proteomics, Biomarkers, Oral cancer

## Abstract

**Background:**

Head and neck squamous cell cancer (HNSCC) is the most common cancer associated with chewing tobacco, in the world. As this is divided in to sites and subsites, it does not make it to top 10 cancers. The most common subsite is the oral cancer. At the time of diagnosis, more than 50% of patients with oral squamous cell cancers (OSCC) had advanced disease, indicating the lack of availability of early detection and risk assessment biomarkers. The new protein biomarker development and discovery will aid in early diagnosis and treatment which lead to targeted treatment and ultimately a good prognosis.

**Methods:**

This systematic review was performed as per PRISMA guidelines. All relevant studies assessing characteristics of oral cancer and proteomics were considered for analysis. Only human studies published in English were included, and abstracts, incomplete articles, and cell line or animal studies were excluded.

**Results:**

A total of 308 articles were found, of which 112 were found to be relevant after exclusion. The present review focuses on techniques of cancer proteomics and discovery of biomarkers using these techniques. The signature of protein expression may be used to predict drug response and clinical course of disease and could be used to individualize therapy with such knowledge.

**Conclusions:**

Prospective use of these markers in the clinical setting will enable early detection, prediction of response to treatment, improvement in treatment selection, and early detection of tumor recurrence for disease monitoring. However, most of these markers for OSCC are yet to be validated.

**Supplementary Information:**

The online version contains supplementary material available at 10.1186/s12957-021-02423-y.

## Background

Oral squamous cell cancer (OSCC) is the most common malignant neoplasm arising in the mucosa of oral cavity and includes subsites like the buccal mucosa, alveolus (upper and lower) tongue, palate, and lip [1]. Head and neck cancer accounts for more than 550,000 cases worldwide annually [2]. Oral cancers are more common in the Indian subcontinent, while cancer of the laryngopharynx is more common in other populations [3]. Overall, 57.5% of global oral cancers occur in Asia especially in India. It is 30% of all cancers in India, of which 60 to 80% of the patients present with advanced diseases as compared to 40% in developed countries, this also suggests lack of awareness and need for markers of early identification [4].

Almost all of these malignancies are squamous cell carcinomas (SSCs) which historically in the developed world was associated mostly with alcohol and tobacco consumption and the combination of the two, producing a synergistic increase in the risk. However, over the past 20 years, investigators have found a growing proportion of HNSCC patients with human papillomavirus (HPV) positive tumors that develop in younger people and those having a lower or no intake of tobacco and alcohol, the association in oropharynx is higher than oral cavity [5].

Improvement in understanding the steps leading to carcinogenesis will enable the identification and prediction of malignant progression at an earlier stage of OSCC. Cancer signifies deviation from normal signaling network toward a dysregulated cellular proliferation. Proteins with linkages to various pathways when altered the functional state may shift the equilibrium of the signaling network to enhance the survival of the affected cells or reduce its apoptosis [6]. Searching for such proteins is the main purpose of cancer proteomics. Proteins being the common molecule that participate in the cellular function are often affected by disease, response to treatment, and being disease free. Development of novel protein biomarkers of OSCC in the light of proteomics can help in early cancer diagnosis, treatment, and prognosis.

## Material and methods

This systematic review was performed as per PRISMA guidelines. A bibliographic search was performed for studies published till August 2021, using PubMed, Cochrane database, Google scholar, the National Library of Medicine, SpringerLink, and Science Open. The keywords used were “proteomic biomarkers,” AND “head and neck cancer,” AND “oral cancer.” The detailed search strategy for PubMed is detailed in Additional file 1. All relevant studies assessing proteomic characteristics of oral cancer and precancers were considered for analysis. Abstracts, incomplete articles, and non-comparative studies and article in language other than English were excluded. We performed a restriction of articles including only studies in humans; studies on cell line and animals were excluded. 

The review also discusses proteomics-based techniques that are used in the identification of proteins that are altered in the disease process or in response to treatment or disease stage and course, and such information could be used to individualize therapy. Research findings in the review are highlights from articles focusing on proteomic approaches toward diagnosis and detection of oral cancer; identification of biomarkers through proteolytic analysis carried out using mass spectrometry, 2D electrophoresis, and other proteomic techniques.

## Results

**Fig. 1:**
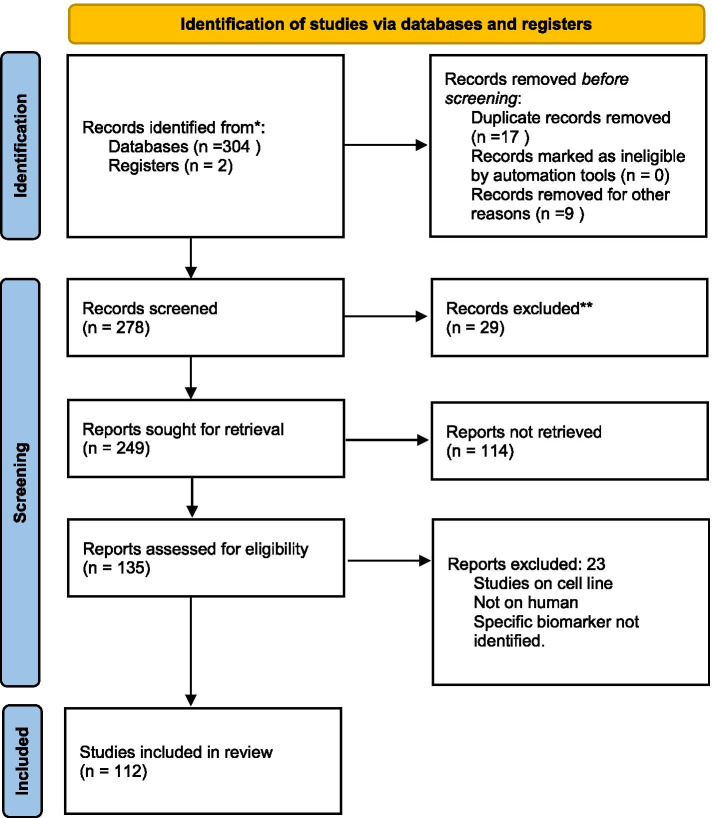
PRISMA flowchart of studies included in the systematic review

The search revealed 304 articles in English of these this systematic review includes a total of 112 articles (Fig. 1). The review articles were excluded, the two meta-analyses published on the subject has been discussed. These articles were categorized under subsections enumerated below followed by a list of all protein biomarkers identified and brief description of their importance.

### Biomarker discovery—a proteomic approach

A biomarker is “a measurable indicator of a specific biological state relevant to the risk of contraction, presence or the stage of disease.” Biomarkers can be clinically used to screen, diagnose, and monitor the activity of disease and to assess therapeutic response [7]. “An ideal biomarker should be sensitive, specific, cost-effective, and robust against situational variability and should have added value beyond that of current standards [8].”

Biomarkers can be carbohydrates, DNA, mRNAs, proteins, or small molecules like metabolites and other cellular molecules [9]. Predictive biomarkers lead to detection of abnormalities that causes the development of OSCC [10], while prognostic biomarkers help in predicting the response to therapy and prognosis of patient. Nucleic acid-based microsatellite analysis and tumor-specific aberrant promoter methylation have been used as markers to detect tumor-specific alterations in body fluid and somatic cells of patients with OSCC [11, 12], while this article focuses on protein biomarkers.

Genome sequencing has produced a wealth of information during the last two decades. Following this step was taken to look at proteins, which are the biomolecules translated from genes and govern overall cellular processes. It is proposed that the genes exert their actions through proteins to cause diseases including malignancies. Mechanisms like alternative splicing and post-translational modifications of proteins (e.g., phosphorylation, glycosylation, acetylation, and proteolytic cleavage) contribute to the human proteome that comprises more than half a million proteins [13, 14] in comparison with about 22,000 protein-coding genes [15]. Proteins are important cellular molecules that participate in the cellular process and even control synthesis of DNA and its transcription, proteomic techniques can provide greater insight into cellular physiology and molecular biology. Biomarkers are of extreme importance and can be utilized either alone or in combination with other biomarkers. The available tools are able to identify the quality, quantity, and structural modification beside sub cellular localization [16]. However, most of these require validation.

These protein biomarkers can be secreted by tumors and hence could be differentially expressed compared to normal tissue. The fresh tissue is generally required to study the translation while paraffin-embedded tissue can be used for cellular localization and study of expression. Apart from serum, these proteins can be estimated in other body fluids like urine, saliva, sputum, etc.; however, their quantity may vary according to their secretion by the tumor.

Before being analyzed by mass spectrometry (MS), the sample undergoes preliminary separation, enrichment or fractionation of their proteins. The techniques of the enrichments include one-directional polyacrylamide gel electrophoresis (1D-PAGE), two-dimensional polyacrylamide gel electrophoresis (2D-PAGE) among others [17].

Liquid chromatography, coupled to tandem MS (LC–MS/MS) is used to identify and quantify proteins from human tissues. This is based on interactions between protein, peptide, and column. First, the separation is done by liquid chromatography before identification by mass spectrometry (MS).

A mass spectrometer (MS) has mainly three components: an ionization source, a mass analyzer, and an ion detector [18, 19]. The most common ion sources used are electrospray ionization (ESI) and matrix-assisted laser desorption/ionization (MALDI). These sources produce ion from the sample which are then analyzed on mass spectrophotometer. The main ion analyzers used in proteomics are quadrupole (Q), time of flight (TOF), ion traps, and Fourier transform ion cyclotron (FT-ICR). The cellular localization and quantification are usually done by immunohistochemistry and ELISA; these are also used for validation of protein biomarkers.

### OSCC biomarkers

As there is considerable variation in protein expression, there is a variety of potential biomarkers of OSCC. These can be broadly classified in to (i) tissue-based biomarkers, (ii) secretomes (plasma, saliva, blood, or other secretions), and (iii) autoantibodies.

### Potential biomarkers

#### Tissue-based biomarkers in head and neck cancer (Table 1)

Majority of selected biomarkers investigated are tissue-based biomarkers by using different approaches and are summarized in Table 1. The approaches employed include LC-MS, RPLC-MS, SELDI-TOF MS, 2D DGE, iTRAQ, and 2DLC. Further, the results verified by using IHC, PCR, and western blot techniques as described above.

**Table 1:** Potential protein tissue biomarkers of head and neck cancers

Sample	Technique	Biomarker [accession no.]	Confirmation by	Biomarker for	Discussion	Reference
OSCC	RPLC-MS/MS: MS	Desmoglein-3 [P32926]	IHC	Carcinogenesis	Desmoglein 3 maintains structural integrity preferentially in oral epithelium. Changes in their relative levels might represent putative biomarkers of disease progression.	[18]
OSCC	RPLC-MS/MS: MS	Cytokeratin 4 [P190013]	IHC	Carcinogenesis	Expressed predominantly in suprabasal-nonkeratinizing layer of stratified epithelium of control normal tissues, whereas OSCC was restricted to only few well differentiated tumors.	[19–22]
OSCC	RPLC-MS/MS: MS	Cytokeratin 16 [P08779]	IHC	Carcinogenesis	Present in suprabasal layer of oral squamous epithelium in the normal tissue, whereas OSCC expresses positive in most moderate to well-differentiated cells	[18, 20, 23, 24]
OSCC	RPLC-MS/MS: MS	Desmoplakin [P15924];	IHC	Carcinogenesis	Predominantly membranous with high immunoreactivity in suprabasal areas of squamous epithelium in normal tissues, whereas OSCC expresses dominantly along the well-differentiated areas of tumor cells	[25, 26]
OSCC	RPLC-MS/MS: MS	Vimentin [P08670]	IHC	Carcinogenesis	Involved in epithelial-mesenchymal transition. Malignant squamous cells in tumor cells show high immunoreactivity to vimentin as in contrast to a few isolated normal cells.	[18, 26, 27] ,
OSCC	IMAC30 protein arrays; SELDI-TOF MS	α-Defensins 1-3 [DEF1-3]	Tissue microarray; IHC	Tumor relapse	Major constituent of azurophilic granules of neutrophils. Normally do not express in epithelia. Plays vital role in mucosal innate immune defense to infectious diseases including epithelial cancers. In OSCC, increased expression of α defensin 1-3 has been described in neutrophils that infiltrate OSCC. In healthy mucosa defensin expression is limited to submucosal neutrophil granulocytes. Represents an important link between inflammation, angiogenesis, and cancer.	[25]
OSCC	2D DIGE	Keratin 4 [P19013]	IHC; ROC; KMS	OSCC premalignant tissue and second field cancer tissue Prognostic	Low expression of keratin 4 in resection margins of surgically treated OSCC patients accurately predicts local relapse. Loss of keratin 4 expression is a valuable enrolment criterion for tertiary prevention trials in treated OSCC patients.	[19, 21, 28]
OSCC	2D DIGE	Keratin 13 [P13646]	IHC; ROC; KMS	OSCC premalignant tissue and second field cancer tissue	Keratin 13 is a protein involved in differentiation process, expression of which changes during the carcinogenic process. Keratin 13 and keratin 4 are such dimers that aggregate to form intermediate filaments of cytoskeleton in epithelial cells.	[19, 21]
OSCC	2D DIGE	Cornulin [Q9UBG3]	IHC; ROC; KMS	OSCC premalignant tissue and second field cancer tissue	Loss of expression in the surgical margin predicts the risk of local relapse.	[19]
OSCC	2D DIGE	Small proline-rich protein 3 [Q9UBC9]	IHC; ROC; KMS	OSCC premalignant tissue and second field cancer tissue	Expresses high in normal mucosa and low in tumors. Belongs to protein group forming cornified envelope, which is an important protective barrier of mucosa and skin and is involved in the differentiation process.	[18]
OSCC	2D GE iTRAQ/MDLC	Stratifin [P31947]	IHC WB; rt-PCR; Co-immunoprecipitation assays; ROC; KMS	OSCC	Overexpressed in HNSCC. Stratifin protein recognizes phosphoserine/threonine-containing motifs to bind target proteins that play important roles in regulation of various cellular processes, including regulation of oncogenes and tumor suppressor genes in carcinogenesis	[18, 25, 29–32]
OSCC	iTRAQ/MDLC	YWHAZ 14-3-3 zeta/delta [P63104]	IHC; WB; rt-PCR; Co-immuno precipitation assays; ROC; KMS	OSCC	Overexpressed in different stages of development of OSCC. Involved in cell signaling pathways in inflammation, cell proliferation and abrogation of apoptosis during oral carcinogenesis. Stratifin-YWHAZ heterodimer may serve as a plausible therapeutic strategy by using a small molecule modulator/peptide inhibitor that intervenes with 14-3-3 client protein interactions.	[29, 30]
OSCC	iTRAQ/MDLC	S100-A7 [P31151]	IHC; WB; rt-PCR; Co-immuno precipitation assays; ROC; KMS	Prognostic	A calcium-binding protein, originally identified in psoriatic keratinocytes and is upregulated in abnormally differentiated keratinocytes. Also identified in oral premalignant epithelium and is proposed to be a marker for invasion.	[29, 33–38]
Oral premalignant (leucoplakia)	iTRAQ	hnRNPK [P61978]	IHC; WB; rt-PCR; ROC	Epithelial dysplasia (leukoplakia)	Heterogeneous nuclear ribonucleoprotein K is an RNA-binding protein that regulates gene expression at both transcriptional and translational level. It directly regulates the expression of COX2, implicated in the synthesis of prostaglandins, which are mediators of inflammatory response. hnRNPK is overexpressed aberrantly localized, whose transcriptional upregulation as reported in OSCC.	[39–41]
Oral premalignant (leucoplakia)	iTRAQ	PTHA [P06454]	IHC; WB; rt-PCR; ROC	Epithelial dysplasia (leukoplakia)	Prothymosin alpha, overexpressed in oral premalignant lesions, proposed to be a proliferation marker of thyroid cancer.	[29, 42]
OSCC	^16^O/^18^O-labeling; 2DLC	Thymidine phosphorylase [TYPH] [p19971]	WB; IHC	Tissue markers for OSCC	TYPH is overexpressed in wide variety of solid tumors and can be induced by several cytokines and contributes to angiogenesis.	[39, 40]
OSCC	^16^O/^18^O-labeling; 2DLC	Filamin-A [P21333];	WB; IHC	Tissue markers for OSCC	Filamin A is involved in organization of extracellular matrix that assists the exchange of signals. Overexpressed in OSCC and is reported as a target for DNA-damage based cancer therapy.	[39, 43]
OSCC; OSCC	^16^O/^18^O-labeling; 2DLC	Fascin [Q16658]	WB; IHC	Tissue markers for OSCC	Fascin is a globular actin-cross-linking protein that forms parallel actin bundles in cell protrusions. Fascin overexpression promotes cancer progression via AKT and MAPK pathways in OSCC.	[39, 44]
OSCC	^16^O/^18^O-labeling; 2DLC	Carbonic anhydrase 2 [P00918]	WB; IHC	Tissue markers for OSCC	Carbonic anhydrases catalyze the equilibrium of carbon dioxide and carbonic acid. Found to be overexpressed in OSCC and can be used to predict local tumor growth in OSCC.	[39, 45]
OSCC	2D GE; Coomassie	Rack1 [P63244]	WB; IHC; RT-PCR; Rack1 si-RNA	Severe dysplasia	Originally identified as anchoring protein for protein kinase C, highly conserved among all eukaryotes and linked to translation initiation in all organisms. Overexpression of RACK 1 in OSCC cancer cell lines suggests potential oncogenic property for RACK1 in oral carcinogenesis.	[45]
OSCC	LC-MS	Keratin 13 [gi62897663]	WB; IHC; rtPCR;	OSCC	Cancer demonstrated down regulation of keratin 13 in OSCC. Aberrant expression indicates dysregulation and cellular transformation of epithelial cells in OSCC.	[46]
OSCC	LC-MS	Keratin 4 [109225249]	WB; IHC; rtPCR;	OSCC	Keratin 4 expression was found to be significantly decreased in OSCC samples. Low expression of keratin 4 is associated with morphological changes in affected oral epithelium and can cause changes in cell shape and movement.	[46]
OSCC	LC-MS	Transglutaminase 3 [gi80478896]	WB; IHC; rtPCR	OSCC	Significantly down regulated in cancer and correlated with loss of histological differentiation. Reduction in transglutaminase 3expression is related with dedifferentiation, increase in invasive phenotype and poor prognosis.	[46]
OSCC tongue cancer	LC-MS	Annexin I [gi442631]	WB; IHC; rtPCR	OSCC Carcinogenesis	Glucocorticoid-inducible protein, Annexin I has emerged as an important endogenous modulator of inflammation. Evident downregulation of ANXA1 in the cancerous lesions was observed.	[46–49]
OSCC	2D GE	Enolase1 [P06733]	IHC; rt-PCR	OSCC	Glycolytic enzyme present in cytoplasm, acts as plasminogen receptor on the surface of cells. Detection of enolase 1 was observed significantly higher in OSCC patient saliva compared to healthy individuals. Useful as biomarker for OSCC.	[50]
Tongue cancer	2D-DIGE	Cofilins	nLC-MS/MS	Carcinogenesis	Actin binding protein participate in cellular motility severing actin filament, nucleating, depolymerizing, and bundling activities	[47, 51]
Tongue cancer	2D-DIGE	Myosin light chain family members	nLC-MS/MS	Carcinogenesis	Myosin light chains are members of the calmodulin (CaM) and CaM-related gene families involved in the mechanoenzymatic function of the myosin holoenzyme	[47, 52]
Tongue cancer	2D-DIGE	Annexin 5	nLC-MS/MS	Carcinogenesis	Member of the calcium and phospholipid binding protein family act as immune check point inhibitor and tumor homing molecule	[47]
Tongue cancer	2D-DIGE	HSP A8	nLC-MS/MS	Carcinogenesis	Heat shock cognate 71 act as repressor of transcriptional activator	[47, 53]
Tongue cancer	2D-DIGE	Carbonic anhydrase 1 (CA1)	nLC-MS/MS	Carcinogenesis	Carbonic anhydrases 1 belong to family of zinc metalloenzymes that catalyze the reversible hydration of carbon dioxide	[47]
Tongue cancer	2D-DIGE	HSP5a (HSP70) Heat shock protein family A (HSP 70) member 5	nLC-MS/MS	Carcinogenesis	Post-translational transport of small presecretory proteins across endoplasmic reticulum	[24, 47, 53]
Tongue cancer	2D-DIGE	Serpin B3	nLC-MS/MS	Carcinogenesis	Papain-like cysteine protease inhibitor to modulate the host immune response against tumor cells	[47]
Tongue cancer	2D-DIGE	Tropomyosin alpha-4 chain TPM 4	nLC-MS/MS	Carcinogenesis	Binds to actin filament and participate in muscle contraction along with troponin complex and calcium dependent regulation	[47]
Tumor	IHC	Cystanin B	Targeted proteomics	Prognostic	Cysteine protease inhibitors	[54]
OSCC	iTRAQ	Gelsolin	Immunoassay	Prognostic	Actin-modulating protein that participate in severing, and capping cytoskeletal actin	[55]
OSCC	iTRAQ	Fibronectin	Immunoassay	Prognostic	Extracellular matrix glycoprotein binds to integrins	[55]
OSCC	iTRAQ	Haptoglobin	Immunoassay	Prognostic	Acute phase protein capable of binding hemoglobin	[55, 56]
OSCC	IHC	Prothymosin α (PTMA)	IHC	Prognostic	Major component of Thymosin Fraction 5 enhances cell-mediated immunity in humans	[33]
OSCC	IHC	Heterogeneous nuclear ribonucleoproteinK (hnRNPK)	IHC	Prognostic	Binds to pre-messenger RNA as a component of heterogeneous ribonucleoprotein particles controls cell cycle progression	[33]
OSCC		Rho GDP-dissociation inhibitor alpha (RhoGDIα)		Prognostic	Regulates the GDP/GTP exchange reaction of the Rho proteins by inhibiting the dissociation of GDP and binding of GTP	[31, 57]
OSCC	2D-PAGE/MS	Annexin A2	MS	Carcinogenesis	Involved in cell motility, linkage of membrane-associated protein complexes to the actin cytoskeleton, endocytosis, fibrinolysis, ion channel formation, and cell matrix interactions	[58]
OSCC	MS	Complement component C7	MS	HPV-induced carcinogenesis	Membrane attack complex (MAC) protein that plays a role in the innate and adaptive immune response	[59]
OSCC	MS	Apolipoprotein F (ApoF)	MS	HPV-induced carcinogenesis	Sialoglycoprotein resides on the HDL and LDL fractions of plasma	[59]
OSCC	MS	Galactin 3-binding protein	MS	HPV-induced carcinogenesis	Glycoprotein induced by inflammatory cytokines and interleukins	[59]
OSCC	AMIDA	Cytokeratin 8		Carcinogenesis	One of the keratin proteins associated with secretary epithelium	[60]
OSCC	2D-GE	Heat shock protein 60	MS	Carcinogenesis	Also known as chaperonin prevent misfolding of proteins	[61]
OSCC	2D-GE	Heat shock protein 27	MS	Carcinogenesis	Also known as HSP B1 participate in thermotolerance, inhibition of apoptosis, regulation of cell development, and cell differentiation	[61]
OSCC	GeLC-MS/MS	Nidogen 1 (NID1)	IHC	Carcinogenesis	Known as entactin is a component of the basement membrane	[62]
OSCC		Thrombospondin 2 (THBS2)		Prognosis	Mediates cell-to-cell and cell-to-matrix interactions	[63]
OSCC	IHC	End-binding protein (EB1)	IHC	Carcinogenesis	Regulate microtubule dynamics	[64]
OSCC	IHC	S100 A2	IHC	Carcinogenesis	Induced by p53, contribute to transcription of p21, differentiation and regeneration	[65, 66]
OSCC	IHC	Scara5	IHC	Carcinogenesis	Act as ferritin receptor found in many cancers	[67]
OSCC	IHC	S100 A8	IHC	HPV-induced carcinogenesis	Calcium- and zinc-binding protein regulator of inflammation and modulate immune response	[68, 69]
OSCC	IHC	Galactin 7	IHC	Carcinogenesis	β-galactoside sugar 15 different types are known	[70]
OSCC	IHC	Survivin	IHC	Carcinogenesis	Inhibitor of apoptosis protein	[71]
OSCC	IHC	SCC antigen	IHC	Carcinogenesis	Tumor-associated antigen present in cases of SCC	[71]
OSCC		Keratin 1		Carcinogenesis	Expressed in epidermis interaction with other genes is known	[48]
OSCC	IHC	P53	IHC	Carcinogenesis	Protein product of tumor suppressor gene, participate in DNA repair	[72]
OSCC	IHC	Deleted in liver cancer (DLC1)	IHC	Carcinogenesis	Protein product of tumor suppressor gene regulates Rho GTPase-activating protein (GAP) domain	[73]
OSCC	IHC	Carcinoembryonic antigen-related adhesion molecule 1 (CEACAM1)	IHC	Carcinogenesis	Mediates cell adhesion by homo and heterophilic bindings	[74]
OSCC	2DE	Proteosome activator PA28 a, b and g	MS	Carcinogenesis	Protein activator of the 20 S proteasome	[75]
OSCC	MALDI TOF	NCOA7	IHC	Carcinogenesis	Nuclear receptor coactivator 7 enhances transcriptional activities and coactivate several other nuclear receptors	[76]
OSCC	IHC	C6orf141 (chromosome 6 open reading frame 141)	IHC	Carcinogenesis	Cell membrane protein found in many cancers, associated with prognosis of breast and endometrial cancer.	[77]
OSCC	IHC	SOD2 superoxide dismutase 2	IHC	Lymph node metastasis	Member of the iron/manganese superoxide dismutase family	[78]
OSCC	IHC	BST2 bone marrow stromal cell antigen 2	IHC	Lymph node metastasis	Acts as a direct physical tether, holding virions to the cell membrane and linking virions to each other.	[78]
OSCC	IHC	ITGB6 integrin subunit beta 6	IHC	Lymph node metastasis	Receptor for fibronectin and cytotactin	[78]
OSCC	IHC	PRDX4 peroxiredoxin-4	IHC	Lymph node metastasis	Member of the peroxiredoxin family of antioxidant enzymes	[78]
OSCC	MALDI	LRP6 low-density lipoprotein receptor-related protein 6	IHC	Carcinogenesis	Coreceptor of Wnt/beta-catenin signaling	[79]
OSCC	Bioluminescence	Lactate dehydrogenase (LDH)	Bioluminescence	Carcinogenesis	Catalyzes the conversion of lactate to pyruvate and back	[80]

*Note*: *IHC* immunohistochemistry, *WB* western blot, *ROC* receiver operator characteristic analysis, *KMS* Kaplan-Meier survival analysis, *iTRAQ* isobaric tags for relative and absolute quantitation, *RP* reverse phase, *OSCC* oral squamous cell carcinoma

Modified from Schaaij-Visser BM. Biomarker discovery for head and neck cancer. A proteomics approach. Ipskamp Drukkers B.V., Enschede, The Netherlands ISBN: 978-90-393-5253-3 [81]

#### Serum/plasma biomarkers/saliva/secretome (Table 2)

Majority of selected serum/plasma-based biomarkers by using different approaches are summarized in Table 2. Only a few important ones are discussed.

**Table 2:** Potential protein biomarkers of head and neck cancers: Serum/plasma/saliva/secretome

Sample	Technique	Biomarker [Accession no.]	Confirmation by	Biomarker for	Discussion	Reference
OSCC	sDIGE&iTRAQ/2DLC	EGFR [A8K2T7]	ELISA; WB; IHC	Carcinogenesis	High EGFR expression is observed in OSCC suggesting that an uncontrolled growth may be mediated by abnormal EGFR expression.	[72, 82]
OSCC	2D-DIGE; MALDI-TOF MS	Vitamin D-binding protein [P02774]	ELISA; WB	OSCC plasma marker	Transport protein for Vitamin D sterols in serum that prevents polymerization of actin. The level of Vitamin D-binding protein level was significantly low in OSCC plasma.	[50, 72, 83]
OSCC plasma saliva	2D-DIGE; MALDI-TOF MS	Fibrinogen alpha chain [P02671]	ELISA; WB	OSCC plasma marker	Plasma fibrinogen is a blood coagulation regulator associated with angiogenic and metastatic prediction in numerous tumors.	[50, 84]
OSCC	2D-DIGE; MALDI-TOF MS	Fibrinogen beta chain [P02675]	ELISA; WB	OSCC plasma marker	Blood-borne glycoprotein, functions in inflammatory responses. Showed elevated expression in OSCC samples.	[50]
OSCC	2D-DIGE; MALDI-TOF MS	Fibrinogen gamma chain [Q9UC63]	ELISA; WB	OSCC plasma marker	Gamma component of fibrinogen has a major function in homeostasis. Can be considered tumor marker, as the protein shows significantly higher expression in OSCC samples compared to the healthy ones.	[50]
OSCC	2D-DIGE; MALDI-TOF MS	Haptoglobin [P00738]	ELISA; WB	OSCC plasma marker	Plasma protein that binds to hemoglobin. Strong correlation was found between increasing levels of haptoglobin and clinical stages of OSCC.	[50, 55, 83]
OSCC	2D-DIGE; MALDI-TOF MS	Leucine-rich alpha-2-glycoprotein/LRG1 [P02750]	ELISA; WB	OSCC plasma marker	This is involved in protein-protein interaction, signal transduction, cell adhesion and development. It is expressed during granulocyte differentiation. The expression was found up-regulated in the disease state.	[50]
OSCC	2D-DIGE; MALDI-TOF MS	RSK2/Ribosomal protein S6 kinase alpha-3 [P51812]	ELISA; WB	OSCC plasma marker	Plays a vital role in cellular and organismal physiology. Activation and elevation of RSK2 is associated with early events of tumorigenesis.	[50, 85–87]
OSCC saliva	Cation exchange/reversed phase LC; 2D GE	S90K/Mac-2 binding protein (M2BP)	ELISA; WB; ROC	OSCC	M2BP, a tumor antigen was significantly up-regulated in nasopharyngeal carcinoma.	[88, 89]
OSCC saliva	Cation exchange/reversed phase LC; 2D GE	S100-A9	ELISA; WB; ROC	OSCC	It is a calcium-binding protein which is significantly overexpressed in saliva of OSCC patients.	[88–90]
OSCC saliva	Cation exchange/reversed phase LC; 2D GE	CD59	ELISA; WB; ROC	OSCC	CD59 is one of the complement restriction factors that are overexpressed on the tumor cells and enable them to escape from complement-dependent and antibody-mediated killing.	[88, 91]
OSCC saliva	Cation exchange/reversed phase LC; 2D GE	Profilin	ELISA; WB; ROC	OSCC	Profilin is regulator of the microfilament system. Overexpressed in tumor cells. Involved in various signaling pathways via interaction with cytoplasmic and nuclear ligand.	[88]
OSCC saliva	Cation exchange/reversed phase LC; 2D GE	Catalase [P04040]	ELISA; WB; ROC	OSCC	Catalase protects the cells against oxidative stress. Altered levels are evident in human tumors and involved in carcinogenesis and tumor progression.	[88]
OSCC saliva	Peptide free flow electro-phoresis; SCX	Signal transducer and activator of transcription 3 [P40763]	WB	OSCC	STAT3 mediates cellular responses to different growth factors. Expression of STAT3 demonstrate its role of development of OSCC.	[92]
OSCC saliva	Peptide Free Flow Electro-phoresis; SCX	Thioredoxin-dependent peroxide reductase, mitochondrial [P30048]	WB	OSCC	Involved in redox regulation of cell. Expression of PRDX3 in whole saliva of patient confirmed the presence of the protein in saliva and can be considered a prospective biomarker of OSCC.	[92]
OSCC saliva	Peptide Free Flow Electro-phoresis; SCX	Serpin B3 [P29508]	WB	OSCC	Modulates the host immune response against tumor cells. Plays tumor inhibitor role.	[92]
OSCC saliva	2D GE	Alpha-1 antitrypsin (AAT)	ELISA; IHC	OSCC	AAT is a serine protease inhibitor. The level of AAT increased in OSCC saliva. Useful for prediction of aggressive phenotypes in OSCC.	[84, 93]
OSCC saliva	2D GE	Complement C3	ELISA; IHC	OSCC	Complement system helps antibodies fight of infections. Gauges the effectiveness of ongoing treatments for autoimmune disorder, cancers and infectious diseases. Level of C2 is proportional to aggression of tumor.	[93]
OSCC saliva	2D GE	Hemopexin [HPX]	ELISA; IHC	OSCC	HPX is plasma protein with highest binding affinity to heme. Distinctive high expression and tumor size parameter shows aggression of cancer.	[93]
OSCC saliva	2D GE	Transthyretin [TTR]	ELISA; IHC	OSCC	TTR is thyroid hormone-binding protein. Overexpressed in non-metastatic OSCC as compared to metastatic.	[51, 93]
OSCC Saliva	MALDI-TOF MS	Zinc finger protein 510 (ZNF510) Zinc finger protein 142 (ZNF142)	IHC; ROC	OSCC	ZNF510 is involved in transcriptional regulation. Found to be expressed in high level in saliva of OSCC patients.	[34, 94]
OSCC Saliva	2D GE; MALDI-TOF	Transferrin	WB; ELISA	OSCC	Transferrin is an iron transport protein whose levels were found elevated in saliva. The level of salivary transferrin shows a relation with size and stage of the disease.	[95, 96]
OSCC saliva	IHC	Lactate dehydrogenase (LDH)	ELISA	OSCC	LDH concentration in saliva indicate cellular necrosis. In an oral OSCC lesion, the level of salivary LDH is expected to increase as the mitotic rate also increases with the aggressiveness of the lesion.	[96]
OSCC saliva	IHC	Cyclin D1 (CycD1)	ELISA	OSCC	CycD1 is a positive regulator of the transition from G1 to S phase in cell-cycle progression. Expression of CycD1 is amplified and over expressed in OSCC.	[96]
OSCC saliva	IHC	Salivary carbonyls	ELISA	OSCC	Protein carbonyls are markers of oxidative stress. Increase in salivary carbonyl points at increased free radical attack. In malignant tissues the degree of oxidative DNA damage is increased whereas total anti-oxidant capacity is decreased.	[96]
OSCC saliva	IHC	Mammary serine protease inhibitor (Maspin)	ELISA	OSCC	Maspin suppress tumor growth and progression, angiogenesis, invasion and metastasis in various malignancies including OSCC.	[96]
OSCC saliva	IHC	8-oxoguanine DNA glycosylase (OGG1)	ELISA	OSCC	Enzyme for repairing the oxidative DNA damage. Reduced activity of OGG1 is a risk factor for various cancers including OSCC.	[96]
OSCC saliva	IHC	Phosphorylated-SRC	ELISA	OSCC	Src (a cytoplasmic kinase) drives adhesion changes that are associated with transition, proliferation and metastasis. It changes to phosphor-Src the inhibited form by oxidants by a reversible process. Src is expected to be increased while phosphor-Src is expected to be decreased to promote carcinogenesis in cancer patients.	[96]
OSCC saliva	IHC	Ki-67	ELISA	OSCC	A cell-cycle promoter that correlate with cellular proliferation, and tumor progression, metastasis and poor prognosis and are expected to increase in tumors.	[96]
OSCC saliva	Immunoradiometric analysis	Carcinoembryonic antigen (CEA)	ELISA	OSCC	A type of glycoprotein produced by cells of gastrointestinal tract during embryonic development and involved in cell adhesion. Salivary and serum levels of CEA were found to be increased in malignant tumors than in healthy tissues.	[97]
OSCC saliva	Immunoradiometric analysis	Carcinoma associated antigen (CA 50)	ELISA	OSCC	A cancer-associated carbohydrate marker. CA50 is not organ-specific and its elevated levels in serum can be seen in variety of malignancies. CA 50 in saliva and serum showed significantly high levels in malignant tumors as compared to the healthy normal tissues.	[97]
OSCC saliva	Radial immunodiffusion	Insulin growth factor-1 (IGF-1)	ELISA	OSCC	IGF plays significant role in carcinogenesis by modifying cancer-cell proliferation, survival, growth and apoptosis. Salivary IGF level was shown to be substantially raised in cancer patients.	[96]
OSCC saliva	Radial immunodiffusion	Metalloproteinase (MMP2)	ELISA	OSCC	Metalloproteinases participates in cancer pathogenesis by degrading type-IV collagen, elastin, and fibronectin. Highly expressed in stromal cells surrounding the invading front of metastasizing tumors and their levels are elevated in tumor endothelium.	[96, 98, 99]
OSCC saliva	Radial immunodiffusion	Metalloproteinase (MMP9)	ELISA	OSCC	Level of MMP-9 showed an increase in OSCC patients.	[34, 96, 99, 100]
OSCC saliva	WB;PCR	CD44 and soluble CD44	ELISA	OSCC	CD44 shows elevation in majority of HNSCC. Distinguishes cancer from benign disease with high specificity.	[27, 37, 98, 101]
OSCC Serum and Saliva	2-D gel electrophoresis	Tetranectin	Liquid chromatography/tandem mass spectrometry	Lymph node involvement	Plasminogen-binding protein with a C-type lectin domain may be involved in packaging molecules for exocytosis	[102]
OSCC Saliva	ELISA	Interleukin 1b (IL1b)	ELISA	Carcinogenesis	Cytokine mediator of inflammatory response	[103–105]
OSCC saliva	ELISA	Interleukin 8 (IL8)	ELISA	Carcinogenesis	Chemoattractant cytokine mediates inflammatory response	[103–107]
OSCC Saliva	ELISA	Mac-2 binding protein (M2BP)	ELISA	Carcinogenesis	Cell-adhesive protein of the extracellular matrix which self-assembles into ring-like structures and binds beta1 integrins, collagens, and fibronectin	[103]
OSCC saliva Plasma	2DE	Apolipoprotein A1 APOA1	MS	Differentiate from leukoplakia	As component of HDL participate in lipid metabolism	[56, 108, 109]
OSCC saliva	2DE	Alpha amylase	MS	Differentiate from leukoplakia	Hydrolyses alpha bonds of large, alpha-linked polysaccharides, such as starch and glycogen, yielding shorter chains thereof, dextrins, and maltose, normally present in saliva	[108]
OSCC saliva	2DE	cystatins	MS	Differentiate from leukoplakia	Cysteine protease inhibitors	[108]
OSCC saliva	2DE	Keratin 10	MS	Differentiate from leukoplakia	Intermediate filament protein of cytokeratin family. Fibrous protein forming the cellular framework	[108]
OSCC saliva	LC-MRM/MS	Alpha-1-acid glycoprotein (A1AG1; AGP1; OMD1)	MS	Carcinogenesis	Inflammatory acute phase reactant	[110]
OSCC saliva	LC-MRM/MS	Alpha-1-antitrypsin A1AT	MS	Carcinogenesis	Protease inhibitor	[110]
OSCC saliva	LC-MRM/MS	Alpha-1-B Glycoprotein	MS	Carcinogenesis	Glycoprotein of unknown function	[110]
OSCC saliva and plasma	MS	Matrix metaaloprotein 1 (MMP1)	MS	Carcinogenesis	Interstitial collagenase and fibroblast collagenase participate in breakdown of extracellular matrix	[111, 112]
OSCC saliva and plasma	MS	Matrix metaaloprotein 3 (MMP3) Stromelysin-1	MS	Carcinogenesis	Interstitial collagenase and fibroblast collagenase participate in breakdown of extracellular matrix. degrades collagen types II, III, IV, IX, and X, proteoglycans, fibronectin, laminin, and elastin	[111]
OSCC saliva	iTRAQ	Complement factor H (CFH)	MS	Carcinogenesis	Regulators of complement activation family	[84]
OSCC saliva	Luminex-based multiplex	Tumor necrosis factor (TNF)		Carcinogenesis	Cytokine used by immune system for cell signaling	[104]
OSCC saliva	Luminex-based multiplex	Vascular endothelial growth factor (VEGF)		Carcinogenesis	Signaling protein that promotes angiogenesis	[104, 107]
OSCC saliva	MS	Actin	MS	Carcinogenesis	Multi-functional protein form microfilaments in the cytoskeleton	[113]
OSCC Saliva	MS	Myosin	MS	Carcinogenesis	Motor proteins that interact with actin.	[31, 113]
OSCC blood	ELISA, IHC MALDI-TOF-MS	C-reactive protein (CRP)	ELISA, IHC, MS	Carcinogenesis	A protein whose concentration increase in response to inflammation—acute phase protein	[52, 114]
OSCC blood	ELISA, IHC	Leucine-rich alpha-2-glycoprotein (LRG)	ELISA, IHC	Carcinogenesis	Acute phase protein	[114]
OSCC saliva	2D-GE	Zinc-alpha-2-glycoprotein (ZAG)	MS	Carcinogenesis	Participate in glycolipid metabolism	[115]
OSCC saliva	2D-GE	Peroxiredoxin-2 (PRDX-2)	MS	Carcinogenesis	Catalyzes the reduction of peroxides	[49, 115, 116]
OSCC saliva	LC-MS/MS	α-2-Macroglobulin-like protein 1	MS	Carcinogenesis	Inhibitor of proteases, role in auto immune diseases	[117]
OSCC saliva	LC-MS/MS	Kininogen -1	MS	Carcinogenesis	Cofactor for the activation of prekallikrein, factor XII and factor XI	[117]
OSCC saliva	SDS-PAGE and MALDI TOF/TOF	Annexin A8	MS	Carcinogenesis	Participate in membrane-cytoskeleton dynamics	[116]
OSCC Saliva	ELISA	IL6	ELISA	Carcinogenesis	Acts as both a pro-inflammatory cytokine and an anti-inflammatory myokine	[118]
OSCC plasma	MALDI-TOF	GIMAP7 GTPase, IMAP Family Member 7	MS	Carcinogenesis	Regulators of lymphocyte survival and homeostasis, GTP Binding	[119]
OSCC plasma	MALDI-TOF	Rabl3 RAB like protein 3	MS	Carcinogenesis	Unknown function, supposed to participate in cellular regulation	[119]
OSCC plasma	MALDI-TOF	Heat shock protein 90 (HSP 90)	MS	Carcinogenesis	Cell cycle control, cell survival, hormone and other signaling pathways	[120]
OSCC saliva	SDS Page	Resistin adipose tissue-specific secretory factor (ADSF) or C/EBP-epsilon-regulated myeloid-specific secreted cysteine-rich protein (XCP1)	LS-MS	Carcinogenesis	Increases production of LDL	[121]
OSCC brush biopsy	MS	Secretory leukocyte protease inhibitor	MS	Carcinogenesis	Anti-microbial and anti-inflammatory, found in saliva, breast milk, etc.	[122]
OSCC serum	ELISA	Guanylate-binding protein 1 (GBP1)	ELISA	Carcinogenesis	Regulation of membrane, cytoskeleton, and cell cycle progression	[123]

*Note*: *IHC* immunohistochemistry, *WB* western blot, *ROC* receiver operator characteristic analysis, *KMS* Kaplan-Meier survival analysis, *iTRAQ* isobaric tags for relative and absolute quantitation, *RP* reverse phase, *OSCC* oral squamous cell carcinoma, *LC-MRM/MS* multiplexed liquid chromatography multiple-reaction-monitoring mass spectrometry

##### Epidermal growth factor receptor (EGFR)

EGFR is an important member of the family of the membrane-bound tyrosine kinase receptors activated in tumor cells of epithelial origin. This receptor regulates cellular growth, proliferation, apoptosis, differentiation, migration, and secretion of certain proteins [94]. High EGFR expression has been observed in OSCC suggesting that an uncontrolled growth may be mediated by abnormal EGFR expression [82, 124].

##### Vitamin D-binding protein

Vitamin D-binding protein is a secreted transport protein which transports the vitamin D sterols in serum and prevents polymerization of actin. The level of vitamin D-binding protein level was significantly low in OSCC plasma. Plasma fibrinogen is a blood coagulation regulator associated with angiogenic and metastatic prediction in numerous tumors [50]. Vitamin D-binding protein has been used as a biomarker for breast cancer, thyroid cancer, and lung cancer [83]. In oral cancer, it has not been found to be increased in human plasma; however, higher concentrations are observed in mouse plasma [83]. Tung et al. (2013) [82] found vitamin D-binding protein to be reduced in OSCC plasma; these results suggested differential regulation in different species.

##### Fibrinogen (alpha/beta/gamma chain)

Plasma fibrinogen is commonly estimated for blood coagulation and is reported as angiogenic and a metastatic predictor in many tumors [50, 84]. The high expression level of serum fibrinogen has been found to be observed in OSCC patients [50]. Fibrinogen beta chain is a blood-borne glycoprotein, functions in inflammatory responses. It has shown elevated expression in OSCC samples [50]. Fibrinogen gamma chain is a gamma component of fibrinogen and has a major function in homeostasis. It can be considered tumor marker, as the protein shows significantly higher expression in OSCC samples compared to the healthy ones [50].

##### Carcinoembryonic antigen (CEA)

CEA is a glycoprotein produced by the cells of gastrointestinal tract during embryonic development and is involved in cell adhesion. The salivary and serum levels of CEA were found to be increased in malignant tumors than in healthy tissues [97]. It has been reported previously that the content of saliva CEA was significantly higher in oral-maxillofacial cancer patients and benign tumor than in normal persons (*P* < 0.01) [97]. Thus, saliva CEA is of guiding significance to a certain extent for identification of malignant and benign tumor, assisting clinical diagnosis and prognosis monitoring of treatment efficacy for cancer [97].

#### Autoantibodies (Table 3)

Majority of selected biomarkers investigated autoantibodies-based biomarkers by using different approaches are summarized in Table 3. Few important ones are discussed herein.

**Table 3:** Potential protein biomarkers of head and neck cancers: autoantibodies

Sample	Technique	Biomarker [accession no.]	Confirmation by	Biomarker for	Discussion	Reference
OSCC saliva	IHC	P53 autoantibody	ELISA; IHC	OSCC	p53 antibodies found only in serum and saliva of patients showing overexpression of p53 in their tumor tissues. Checks for overexpression of p53 protein however do not differentiate between wild and mutant proteins.	[125]
OSCC	MALDI-TOF/TOF-MS	Heat shock protein 70 (HSP70)	MS	Carcinogenesis	Cellular network of molecular chaperones and folding catalysts assists in protein folding process. Identified as early marker and prognostic marker in OSCC	[126]
OSCC	MALDI TOF-MS/2DE	Sideroflexin 3 (SFXN3)	MS	Response to therapy	Mitochondrial serine transporter participate in one-carbon metabolism pathway.	[127]

*Note*: *IHC* immunohistochemistry, *WB* western blot, *ROC* receiver operator characteristic analysis, *KMS* Kaplan-Meier survival analysis, *iTRAQ* isobaric tags for relative and absolute quantitation, *RP* reverse phase, *OSCC* oral squamous cell carcinoma

##### P53 autoantibody

p53 antibodies are found in serum and saliva of patients showing overexpression of p53 in their tumor tissues. This is an easy process as these can be detected from saliva [125].

##### Hsp 70 autoantibody

HSPs are frequently overexpressed in tumor cells. Autoantibodies directed against HSP70 can discriminate the risk condition between healthy and tumor cells. Its level increases from healthy controls to SCC, suggesting that autoantibodies might be used as both early marker and screening risk marker for SCC [126].

## Discussion

Development of OSCC is a multistep process. Field cancerization is one of the hallmark of oral cancer, wherein the whole of the mucosa of the oral cavity and upper aerodigestive tract undergo molecular changes and is susceptible to develop cancer. Change in the protein expression profile can be a manifestation of the field cancerization and hence its identification is an important biomarker to predict risk of development of cancer, second primary or recurrence of OSCC.

Tobacco and alcohol consumption are the major independent risk factors for development of HNSCC that also show synergy when combined [128]. Oral cancer development risk is 3 to 9 times greater in those who smoke and drink than in those who consume neither of the two [6, 128]. The upper aerodigestive tract is first to make contact with the harmful components of tobacco-like aromatic polycyclic hydrocarbon (PAH), nitrosamines, aromatic amines, and aldehydes that are responsible for malignant transformation [129]. The metabolism of chemicals occurs in two phases. In phase 1, reduction and oxidation reactions occur in cytochrome P-450 system, producing reactive and toxic substances. This oxidative stress induces glutathione S-transferase transcription to eliminate the toxic substances [130]. The toxic metabolites produced genetic instability, mutation, and may initiate the carcinogenesis. After the glucuronidation, sulfation, methylation, and conjunction reactions, the toxic agents are inactivated and become hydro soluble, and are excreted [131]. Mutation of *p53* have been found to occur more frequently in tobacco and alcohol uses [132], suggesting that inactivation of *p53* tumor suppressor gene may play an important role in tobacco-induced carcinogenesis.

Infection with human papilloma virus (HPV) is another risk factor specially for oropharyngeal cancer. This dsDNA virus has a 7 kB genome with number of early and late genes that synthesize proteins. Only a subset of more than 100 known HPV subtypes are oncogenic and high-risk types. HPVs encodes E6 and E7 oncoproteins that inactivates p53 and Rb respectively, leading to failure of tumor suppressor mechanism [133]. Few HPV-associated biomarkers have also been identified.

Association of OSCC with genetic polymorphisms in genes encoding human enzymes related to toxic substance metabolism has also been reported [134] that affects the individual’s susceptibility to noxious effects of cancer. Patients with Fanconi anemia (FA) are predisposed to develop OSCC [135]. Fanconi anemia is a recessive genetic disorder caused by biallelic mutation in a member of FA/BRCA pathway [136]. These cancers usually develop at a young age [137]. Another predisposing factor for cancers of hypopharynx is Plumer-Vinson (also called Paterson-Kelly) syndrome, which results from iron-deficiency [138].

Arroyo et al. [139] in a recent meta-analysis found 11 biomarkers of which they did meta-analysis for 4. Of these, only carcinoembryonic antigens (CEA) and soluble fragment of cytokeratin 19 (CYFRA21) were found to be significantly associated with oral cancer. Kasradze [140] in their review found 44 relevant proteins. Of them, proteins (14-3-3γ, extracellular matrix metalloproteinase inducer, and PA28γ) were found to be most significant. Other studies reported only the number of proteins differentially expressed without any identification [141–144]. Li et al. [145] identified differential protein expression in oral cancer patients with or without lymph node metastasis. Levels of PF4V1 and F13A1 correlated with number of lymph nodes. Immunoglobulin (Ig) Kappa chain C region and Isoform 2 of fructose bisphosphate aldolase A are found to increase in tobacco users; however, these markers are not yet validated [146]. Other investigators found Serpin family of proteins to be overexpressed in tobacco users [147], while some just reported number of proteins with differential expression [148].

The OSCCs occur as a consequence of proto-oncogene activation or tumor suppressor gene inactivation. Promoter hypermethylation is an example of indirect mechanism [149]. The three main alterations in gene function that occur in OSCC are (1) inactivation of p53 tumor suppressor gene, (2) inactivation of cyclin- dependant kinase (CDK) inhibitor p16, and (3) overexpression of epidermal growth factor receptor (EGFR); however, mutations in the EGFR genes occur with very low frequencies.

### Inactivation of p53 tumor suppressor gene

**Fig. 2:**
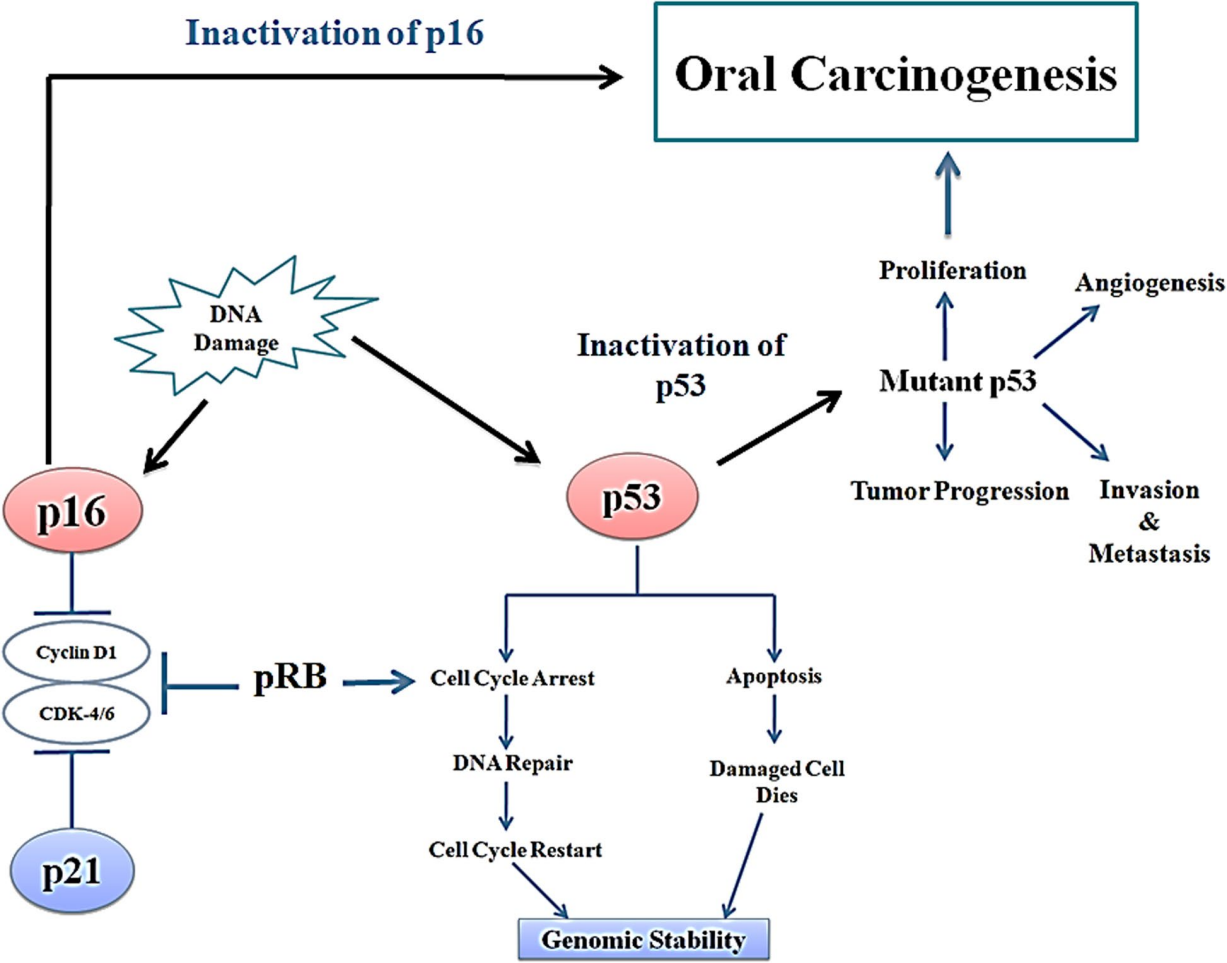
Line diagram showing p53-mediated downstream signaling pathway

p53 has a role in maintaining genomic stability, cell-cycle progression, cell differentiation, DNA repair, and apoptosis, and hence is aptly called the “guardian of the genome.” Mutations, deletions, and binding with viral proteins can produce p53 dysfunction [150]. It is found in approximately 50% of OSCC tumors and is one of the most common cancer development events [151] (Fig. 2).

### Inactivation of cyclin-dependant kinase (CDK) inhibitor p16

CDK are important molecules responsible for regulation of the cell-cycle. A number of these proteins have been identified and some of these can be targeted.

The function of CDK is regulated by number of genes like p16 and retinoblastoma gene. The effect is brought by regulating the phosphorylation of genes during G1 to S phase, through inhibition of CDK 4 and 6 [152]. The formation of CDK 4-6/cyclin D complex is inhibited by the p16 gene, and p21 gene (Fig. 1) thus leading to cell cycle arrest. Downregulation of these proteins is often associated with OSCC [153]. Regulation of phosphorylation of retinoblastoma gene by p16, p21, Cyclin D, and CDK leads to cell cycle arrest, DNA repair, and apoptosis if repair fails (Fig. 1).

### Overexpression of EGFR

EGFR promotes epidermal cell growth and regulates cell proliferation, while regulation of metastasis and angiogenesis leads to development of OSCC. Therefore, EGFR proteins overexpression leads to increased tumor proliferation. EGFR ligand binding results in a molecular cascade that covers receptor-linked tyrosine kinase activation and other downstream pathways. EGFR family has four types of receptors and can have homo or heterodimers where in two similar members or different members bind to produce a dimer. EGFR controls many pathways however its overexpression is found to be associated with increased carcinogenesis [98, 127, 153, 154]. However, for targeting its mutations are normally looked at and mutant EGFR with chromosome 19-21 mutations are often targeted with tyrosine kinase inhibitors.

The biomarker data presented in the article show that this is still a new field and though a lot of the markers are identified, not much work has been done on validating these so far. Further, the data shows differences in the proteomic profile between continents and also between subsites. There is also a difference between tissue and secretome profile wherein more inflammatory markers are seen in saliva. The validation of diagnostic and prognostic biomarkers is a long-drawn process, and there is a need to have more proteomic research to identify better markers that will improve the diagnosis and prognostication of the patients.

## Conclusion

Proteomic and genomic characterization of tumors is essential for identification of biomarkers of carcinogenesis, therapeutics, prognosis, progression, and metastasis. This is frequently been used in many tumors while their role in others is still under investigation. OSCC is uncommon tumor in the west but is common is South East Asia; hence, very little work is done on it. In recent times, the newer evidence has come that shows *p53* and *ras* mutations to be common, and these tumors have poor prognosis compared to that without it. Further work on proteomics will help identify more markers of carcinogenesis, prognosis, and therapeutic significance and will help identify newer targets.

## Supplementary Information

**Additional file 1.** Search strategy

## Data Availability

Not applicable
