# Supplementary material for: A systematic review of proteomic biomarkers in oral squamous cell cancer
Source: World J Surg Oncol. 2021 Oct 28;19:315. doi: 10.1186/s12957-021-02423-y (PMC8555221; doi:10.1186/s12957-021-02423-y)
Supplement: Supplementary file 1 — Additional file 1. Search strategy [file 12957_2021_2423_MOESM1_ESM.docx]

(("proteom"[All Fields] OR "proteome"[MeSH Terms] OR "proteome"[All Fields] OR "proteomes"[All Fields] OR "proteomical"[All Fields] OR "proteomically"[All Fields] OR "proteomics"[MeSH Terms] OR "proteomics"[All Fields] OR "proteomic"[All Fields]) AND ("mouth neoplasms"[MeSH Terms] OR ("mouth"[All Fields] AND "neoplasms"[All Fields]) OR "mouth neoplasms"[All Fields] OR ("oral"[All Fields] AND "cancer"[All Fields]) OR "oral cancer"[All Fields]) AND ("biomarker s"[All Fields] OR "biomarkers"[MeSH Terms] OR "biomarkers"[All Fields] OR "biomarker"[All Fields])) AND ((humans[Filter]) AND (english[Filter]))

Translations

proteomics: "proteom"[All Fields] OR "proteome"[MeSH Terms] OR "proteome"[All Fields] OR "proteomes"[All Fields] OR "proteomical"[All Fields] OR "proteomically"[All Fields] OR "proteomics"[MeSH Terms] OR "proteomics"[All Fields] OR "proteomic"[All Fields]

Oral cancer: "mouth neoplasms"[MeSH Terms] OR ("mouth"[All Fields] AND "neoplasms"[All Fields]) OR "mouth neoplasms"[All Fields] OR ("oral"[All Fields] AND "cancer"[All Fields]) OR "oral cancer"[All Fields]

biomarker: "biomarker's"[All Fields] OR "biomarkers"[MeSH Terms] OR "biomarkers"[All Fields] OR "biomarker"[All Fields]

Filter humans

Total articles : 308

Language English 304
